# Digital interventions for self-management of prediabetes: A scoping review

**DOI:** 10.1371/journal.pone.0303074

**Published:** 2024-05-10

**Authors:** Melanie Stowell, Rosie Dobson, Katie Garner, Mirza Baig, Norma Nehren, Robyn Whittaker

**Affiliations:** 1 National Institute for Health Innovation, University of Auckland, Auckland, New Zealand; 2 Institute for Innovation and Improvement, Te Whatu Ora Waitematā, Auckland, New Zealand; 3 Orion Health, Auckland, New Zealand; 4 Te Hiku Hauora, Kaitaia, New Zealand; University of Montenegro-Faculty of Medicine, MONTENEGRO

## Abstract

**Background:**

Rates of prediabetes, which can lead to type 2 diabetes, are increasing worldwide. Interventions for prediabetes mainly focus on lifestyle changes to diet and exercise. While these interventions are effective, they are often delivered face-to-face, which may pose a barrier to those with limited access to healthcare. Given the evidence for digital interventions addressing other noncommunicable diseases, these may also be effective for prediabetes self-management. The aim of this scoping review was to assess the breadth of evidence around digital interventions for prediabetes self-management.

**Methods:**

We developed a targeted search strategy and relevant studies were identified through searches conducted in four bibliographic databases (Medline, Embase, PsycInfo, and Scopus). Published studies were eligible if they included a digital intervention to support adults aged 18+ with prediabetes self-management. Titles and abstracts were first screened for relevance by one researcher. Full texts of selected records were assessed against the review criteria independently by two researchers for inclusion in the final analysis.

**Results:**

Twenty-nine studies were included, of which nine were randomised controlled trials. Most efficacy studies reported significant changes in at least one primary and/or secondary outcome, including participants’ glycaemic control, weight loss and/or physical activity levels. About one-third of studies reported mixed outcomes or early significant outcomes that were not sustained at long-term follow-up. Interventions varied in length, digital modalities, and complexity. Delivery formats included text messages, mobile apps, virtually accessible dietitians/health coaches, online peer groups, and web-based platforms. Approximately half of studies assessed participant engagement/acceptability outcomes.

**Conclusion:**

Whilst the evidence here suggests that digital interventions to support prediabetes self-management are acceptable and have the potential to reduce one’s risk of progression to type 2 diabetes, more research is needed to understand which interventions, and which components specifically, have the greatest reach to diverse populations, are most effective at promoting user engagement, and are most effective in the longer term.

## Introduction

Prediabetes is a condition referring to the earliest identifiable stage of glucose dysregulation and is used in clinical settings as a mechanism to identify individuals at high risk for developing type 2 diabetes [1]. While diagnostic criteria and definitions for prediabetes may vary [1,2], evidence suggests that as many as 70% of prediabetes cases will progress to type 2 diabetes over the life span [2]. Type 2 diabetes is a progressive disease that is on the rise globally [3] and is characterised by the body’s loss of ability to process insulin. It is associated with a host of health complications, including heart attack, stroke, kidney disease, lower limb amputation, vision loss, nerve damage, and premature death [4]. However, prediabetes can itself be a risk factor for these complications [2]. Treatment of diabetes and its complications can pose a significant economic burden to families and health systems due to the high costs of medical care and loss of wages [4].

Numerous randomised controlled trials (RCTs) have demonstrated the effectiveness of various lifestyle and/or pharmacological methods to prevent or delay the onset of type 2 diabetes [5,6]. Lifestyle interventions have traditionally included dietary advice and group programs designed to increase individuals’ physical activity levels [5]. Some research looking at long-term outcomes has suggested that lifestyle changes can be equally effective as pharmacological treatments in reducing the development of diabetes in the longer term [7]. However, it has been noted that significant behaviour changes are required for diet and exercise interventions to be effective [6]. Similarly, recruitment and retention to in-person programs are limited by factors such as clinic and patient time and resources; patient transportation and access issues; and patient health literacy/language barriers [8,9].

Digital technology now has applications in every aspect of health and healthcare, including digitally mediated diagnostic tools, artificial intelligence, remote patient monitoring and consultation, and consumer-facing mobile health interventions [10]. Digital tools may offer a solution to barriers to in-person diabetes prevention interventions by providing remote education and support for self-management via text messages, applications, and websites [11] and have shown effectiveness in supporting self-management of type 2 diabetes [11–13]. However, there is less evidence regarding the outcomes of digital interventions targeting adults with prediabetes, and more clarity is needed about the specific mechanisms most effective in multi-faceted programs such as those with digital and non-digital components [11]. More evidence is also needed to understand which sub-groups of adults are more or less likely to see positive outcomes from participation in such interventions [11,14], particularly given that underlying disparities in access to care may persist in the digital health sphere [15]. This is especially important in a post-Covid-19 context in which a rapid and necessary transformation to digital health service provision has highlighted the need for more evidence about best practices to achieve value, equity, and quality of services in this sphere [16]. Furthermore, emerging evidence has found that infection with Covid-19 may aggravate metabolic conditions such as prediabetes and type 2 diabetes [17]. Providing all individuals at high risk for diabetes with efficient, effective support has never been more critical.

The aim of this scoping review was to assess the current breadth of evidence about digital interventions designed to promote self-management and improve health outcomes among adults with prediabetes.

## Methods

We employed scoping review methods for this study, as they permit a broad mapping of existing evidence and identification of gaps in the research about a topic [18,19]. Our methods are reported in accordance with the Preferred Reporting Items for Systematic Reviews and Meta-Analyses extension for Scoping Reviews (PRISMA-ScR) S1 Checklist [20] and the protocol was not published.

### Search strategy

A targeted bibliographic database search was developed with input from clinical and information specialists. Searches were conducted on 16 December 2022 using OVID Medline, Embase, PsycInfo, and Scopus. Searches were limited to papers published in English. Search strings were adapted to each database as appropriate; those applied to Medline can be found in S1 Table. Reference lists of relevant previous reviews and included studies were searched for additional papers. Results were managed using Endnote 20 [21].

### Eligibility criteria

Studies were eligible if they included a digital intervention designed to promote self-management of prediabetes and targeted participants aged 18+ years meeting clinical criteria for prediabetes. As clinical diagnoses for prediabetes differ internationally [1,22], no standard definition was applied across the studies. We instead relied on each study’s definition of prediabetes. Studies involving participants with gestational, type 1 or 2 diabetes were included if results for participants with prediabetes were reported separately or were discernible in the results. All study designs were included with the exception of protocols and reviews. Studies published prior to December 2022 were eligible. There were no restrictions on comparator or outcome measures. Only full-text articles published in peer-reviewed journals were included; studies published only in the form of conference abstracts were excluded. Studies were excluded if published in languages other than English. S2 Table summarises the review criteria according to the PICOS framework.

### Study selection

Records were managed using Rayyan, an online platform to facilitate screening for systematic reviews [23]. Titles and abstracts were first screened for relevance by K.G. Full texts of selected records were assessed against the review criteria independently by K.G. Ten percent of articles were screened independently by R.D. at each stage to test application of inclusion criteria; disagreements between reviewers were discussed and resolved.

### Data extraction and synthesis

Relevant data were extracted intro a structured form including author(s), year, aims, country of publication, study design, participant/population characteristics, intervention/control descriptions, and key outcomes/findings. Results were compared and summarised using a descriptive narrative synthesis methodology.

### Quality assessment

Consistent with scoping review methods, our aim was to provide an overview of the existing evidence rather than a critically appraised and synthesised summary [18,19]. A quality assessment was therefore not conducted in this study.

## Results

**Fig 1:**
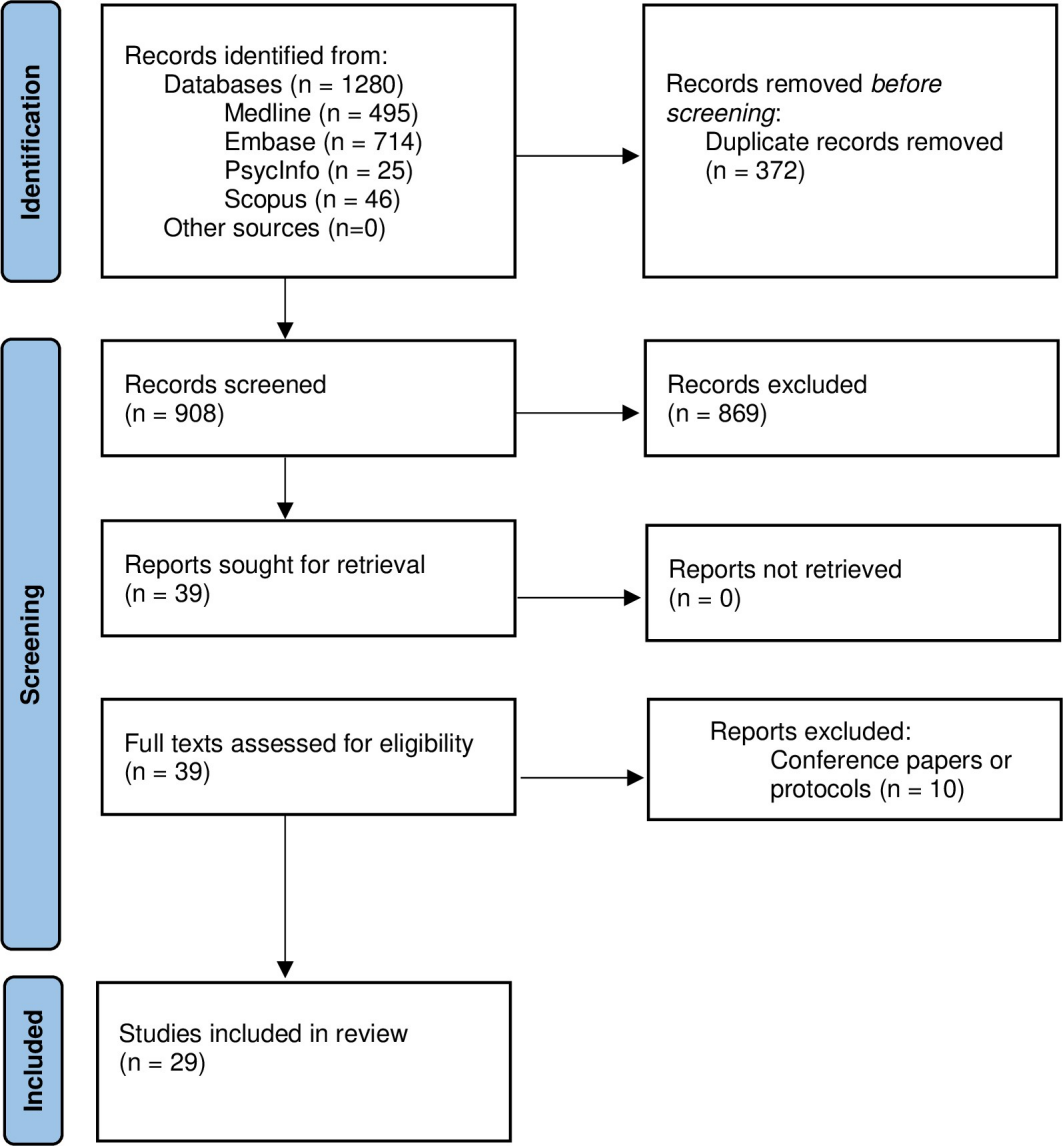
Preferred Reporting of Items for Systematic Reviews and Meta-Analyses (PRISMA) flow chart.

A total of 29 studies met our review criteria (Fig 1) [24–52]. S3 Table presents an overview of included studies; key characteristics are further summarised below.

### Study methods

**Fig 2:**
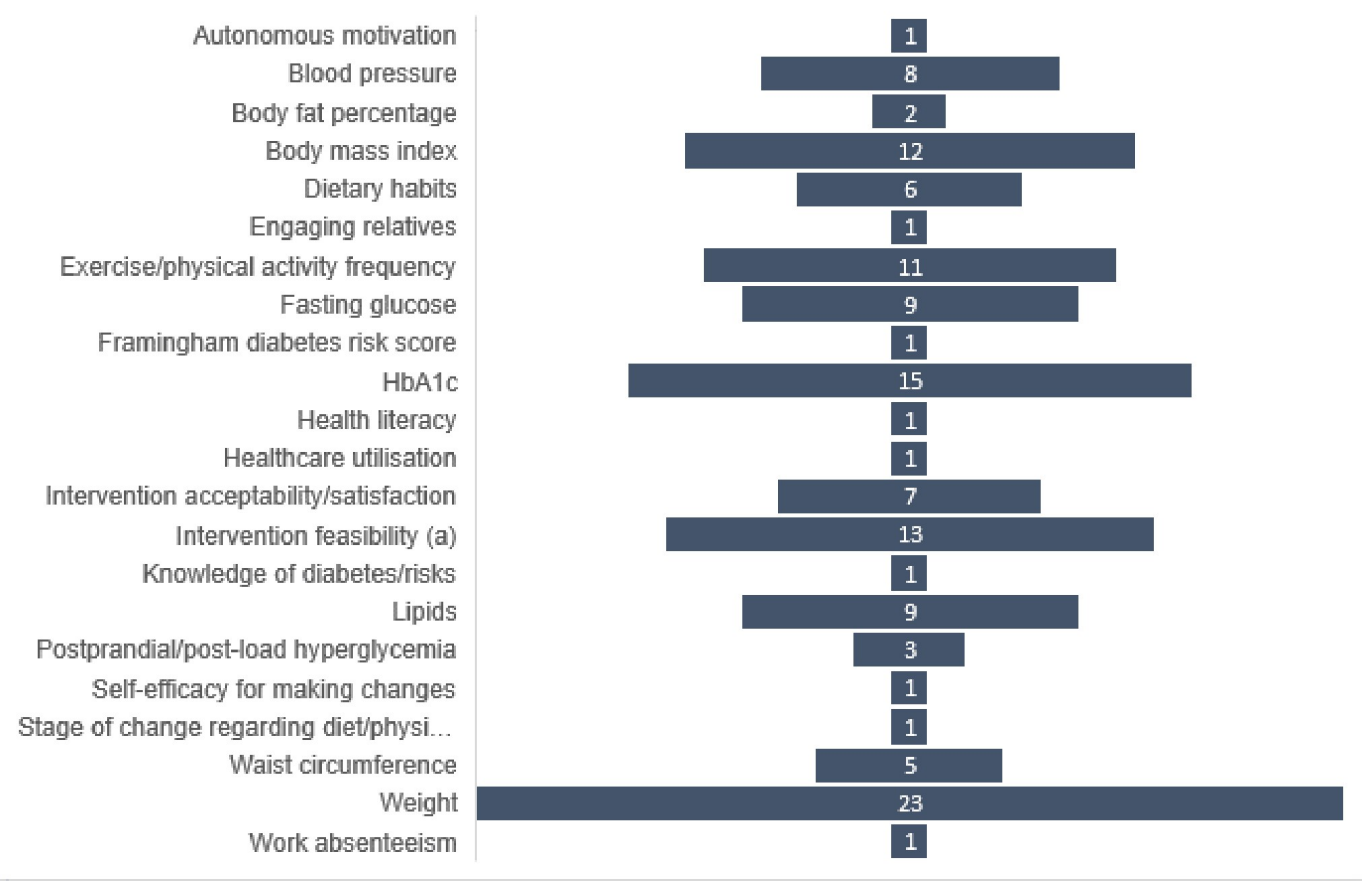
Frequency of primary and secondary outcomes reported by studies. (a) e.g. intervention uptake, retention, engagement, adherence, cost.

Of the studies included in this scoping review, nine were RCTs [27,28,34,38,40,42,43,49,51], six were pilot studies [29–31,37,41,52], nine were quasi-experimental studies [24,32,33,35,39,44–46,50], three were observational/secondary analysis studies [25,26,36], one was a process evaluation [47] and one was mixed-methods [48]. Sample sizes ranged from 18 [48] to 2062 [43]. Follow-up periods ranged from three months [30–32,37,46,48] to three years [45]. Some studies discussed elements of intervention co-design, such as seeking participant input on intervention modalities and content [28,33,34,39,43]. Frequency of primary and secondary outcomes specified in studies are presented in Fig 2. Participant changes in weight, HbA1c, physical activity level, and BMI were the most frequently reported outcomes to measure intervention efficacy.

### Participant characteristics

Approximately half the studies in our review were conducted with adult participants in the United States [27,31–39,41,44,45,51]. All studies were conducted to support individuals diagnosed with or meeting one or more clinical criterion for prediabetes. When specified, this was most frequently measured by haemoglobin A1c (HbA1c) [30,32–35,37–39,41–43,46,47,49], but also prediabetic fasting glucose [24,28–30,32,39–41,46,52], postprandial/glucose tolerance [30,32,39,40,46,52], elevated body mass index (BMI) [32,34,35,38–40,44–46,49], or prediabetes criteria published by national health associations/agencies [36,48,51]. All studies either explicitly required or assumed participant access to the technology featured in the intervention, such as internet, telephone, and/or smartphone, except for one of the interventions targeting healthcare practitioners [29].

### Digital intervention characteristics

Table 1 presents a summary of the types of digital interventions reported. Some studies assessed multimodal interventions or standard care plus a digital intervention: for example, digitally delivered content plus in-person dietitian support [30,46], use of a digital bodyweight scale [25,32,36–40,44,45], a wearable activity tracker [25,26,31,37,38,41,44,45,49], or medication [46]. Some interventions utilised artificial intelligence [36], machine learning [32], or algorithms [27,37,40,50] to deliver tailored digital content to participants based on their progression toward the target outcomes. Pre-emptive tailoring of content to participants’ cultural, language, or literacy needs was also mentioned in some studies [33,34,39,43,50].

**Table 1:** Digital intervention delivery methods.

Delivery method	Frequency^a^
Online / web-based program	13 [27,29,38,39,41,42,44–50]
Virtually accessible, human dietitian/health coach	13 [25,26,30,34,38–40,42,44–47,51]
Mobile app	12 [24–27,30,32,36,37,40,42,47,51]
Social media / online group forums	10 [24–26,38,39,42,44,47,50,51]
Text messages	6 [28,33,34,43,49,52]
Interactive voice response (IVR) phone calls	2 [27,31]
Electronic medical record	1 [41]

^a^Not mutually exclusive where multiple studies reported separate analyses on the same intervention or assessed multimodal interventions.

**Fig 3:**
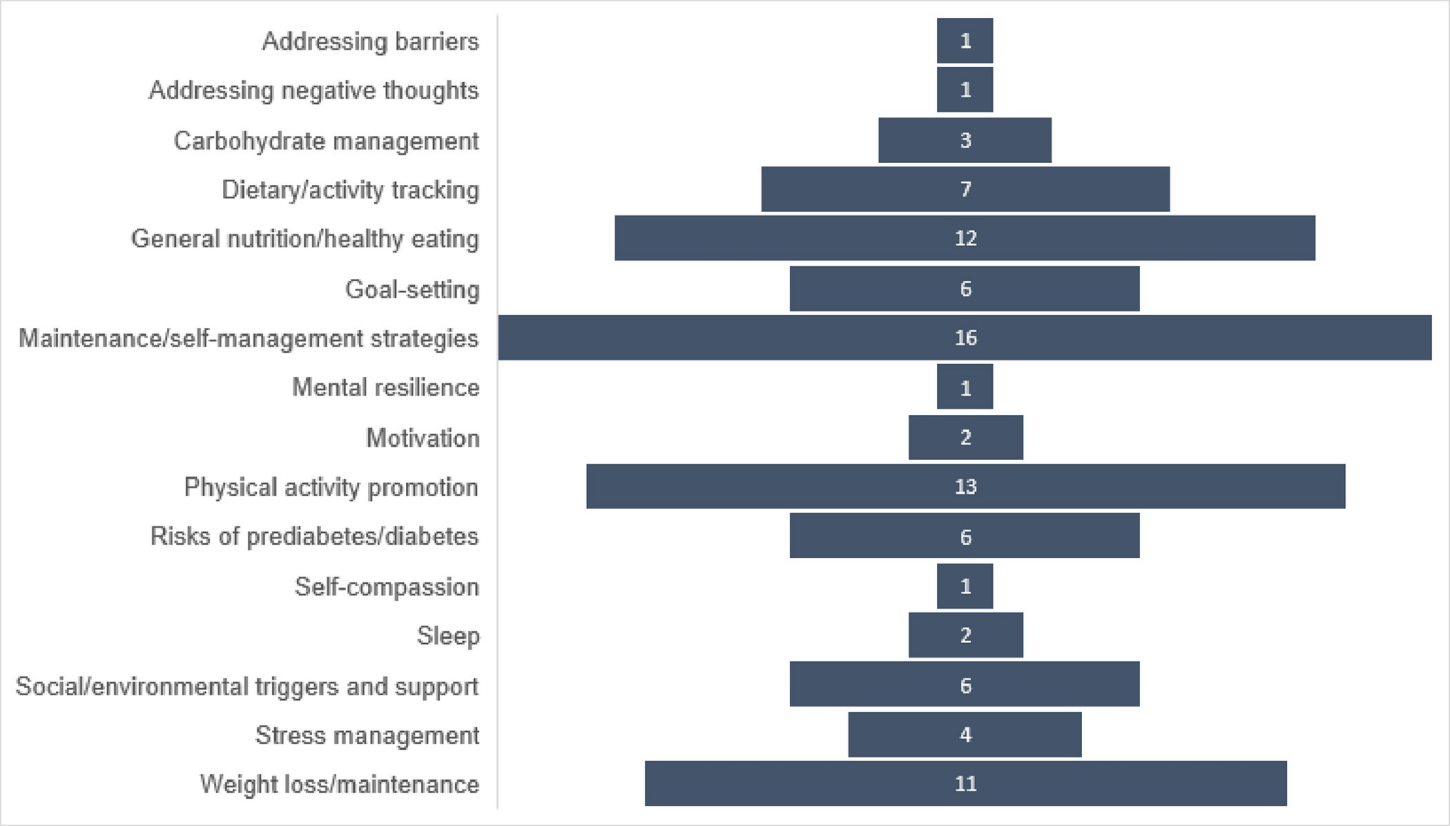
Frequency of topics specified in studies.

Frequency of topics mentioned by studies is summarised in Fig 3. Over one-third of studies [25–27,33–36,38,39,44,45,49,51] tested a digital version of the Centers for Disease Control and Prevention’s (CDC) Diabetes Prevention Program, which consists of a 16-week core curriculum focusing on topics such as healthy eating, physical activity, and social triggers, followed by eight months of maintenance programming [25]. Others described theories or strategies underpinning the digital interventions, including motivational interviewing [29,34,40,42,49,51]; theory of planned behaviour [27,49,52]; behavioural change theory [32,47]; Specific, Measurable, Achievable, Relevant and Time-bound (SMART) goal setting [41,48]; stages of change [41,43]; social cognitive theory [27,52]; behavioural economics [27]; cognitive restructuring [40]; and positive psychology [27].

### Efficacy outcomes

Most efficacy studies in this review reported significant changes in at least one primary and/or secondary outcome. Among the outcomes most frequently reported, studies described significant intervention-related improvements to intervention participants’ weight [24–27,29,30,34,36,38,40,44,45,51], BMI [25,27,29,30,39,51,52], HbA1c [27,35,38,40,44,45,50], and physical activity level [25,26,28,30,41] at follow-up. About one-third of studies reported mixed outcomes or early significant outcomes that were not sustained at long-term follow-up [24,28,32,34,35,40,41,46,48,51,52]. Four studies reported no significant changes among intervention participants [33,42,43,49]. These studies had longer follow-up time points, ranging from six to 24 months.

Of the nine RCTs included in this review, two-thirds reported significant outcomes as a result of the intervention. Compared to control participants, intervention participants in these studies showed significant improvements in weight [27,34,38,40,51], BMI [27,51], HbA1c [27,38,40], and physical activity levels [28].

### Feasibility and acceptability outcomes

Amongst the 15 studies assessing intervention feasibility and/or acceptability [29,32–35,37,39,40,42–48,51], methods to assess these varied and results were mixed. For instance, one study reported a retention rate of 91% after 12 months of a text messaging intervention (defined as those who did not explicitly drop out) [33], while another reported that one-quarter of enrolled participants in a multimodal online program did not engage at all during the 16-week intervention (based on program usage data) [47]. Some studies collecting qualitative feedback described barriers to engagement such as difficulties using a platform, lack of time, lack of interest, and lack of internet access [46]. Positive qualitative feedback from participants included the opportunity to interact with peers with similar experiences, lifestyle changes that felt feasible, and positive perceptions of intervention content [48].

## Discussion

This research provides an overview of current evidence about digital interventions to promote health and support self-management among adults with prediabetes. This review is an important contribution to the literature as the majority of reviews in this area were published prior to the Covid-19 pandemic [11,53–55]; other recent work has had a narrower scope in terms of study population or design [14,56]. Furthermore, more than half of included studies in this review were published in 2020 or later, meaning that the evidence presented is current.

Most studies in this review reported significant positive results in participants’ weight/BMI, HbA1c, and/or physical activity levels, showing the potential of digital interventions for prediabetes. However, non-RCT study designs, intervention complexity, and follow-up time points varied and thus make it difficult to draw firm conclusions about the efficacy of particular digital interventions. For instance, most RCTs were multimodal and shared in common a mechanism to digitally connect participants with a human dietician/health coach [34,38,40,51] or algorithm-tailored content [27] and thus made it difficult to distinguish which elements (automated or human) were most effective. However, the ability to connect with human peers and coaches virtually appears to be an important component in many of these interventions; it is also possible that simply having multiple methods of engagement is sufficient to increase user engagement and satisfaction since it allows participants to engage in the ways most useful to them. The need for more evidence regarding multimodal digital health interventions and their mechanisms of impact has been highlighted by others [57–60]. Recent work assessing the (cost) effectiveness of digital interventions of different modalities to support type 2 diabetes self-management have demonstrated that while cost-effectiveness might be similar across some of the common modalities [61], text message and app-based interventions may be more effective overall and have greater reach than web-based interventions [62]. Future work should seek to address these questions for interventions targeting prediabetic populations.

Half of studies in this review occurred in the United States and a high degree of content was based on the CDC Diabetes Prevention Program (DPP), making this review very US-focused. While most of these studies reported significant positive effects of the digital adaptations of the DPP, it is unclear whether the content would be generalizable to non-US contexts. Only six of the included studies took place outside Asia or North America and most discussed challenges with recruiting diverse samples, representing predominantly female [25,26,31,33,34,39,41,45,46,48,51] and/or racial majority [25,27,31,32,35,36,38,39,47,48,50] participant populations. Having access to the required technology was an explicit or implicit criterion for participation in all studies and most were only offered in one language. More research is therefore needed to understand the feasibility and efficacy of such interventions among more representative populations, where access to technology and cultural considerations may be more variable. A key aspect of digital health is in the potential to provide public health intervention with reach into populations where barriers to existing services exist. Without research studies specifically targeting these groups, arguably the full potential of digital health prediabetes interventions will not be achievable.

Only half of included studies reported engagement data, which is disappointing. Understanding how users engage with digital tools is essential to understanding the effective components. For those that did report on engagement, it was clear that not all participants engaged as expected, and in some cases, there were participants who did not engage at all. There are many barriers to engagement with digital tools, for example technical difficulties, lack of interest, and lack of internet access [45], and ensuring we design tools that minimise the likelihood of these is essential. Further work is also needed to understand how users engage with these tools in real-word settings outside of research trials. It is likely that engagement will be further reduced without controlled research study environments, thus making efforts to increase user engagement in the design even more important.

It was encouraging to see more than half of studies following up with participants at 12 months or later [26,33–36,38,39, 42–45,47,49–52], considering that prediabetes is a long-term condition and sustained behaviour change is unlikely with interventions of short duration. Digital interventions have an advantage over face-to-face interventions in their ability to reach individuals over longer periods of time in a cost-effective manner, thus it is important to continue evaluating the efficacy and sustainability of these programmes with long-term outcomes in mind.

This scoping review had several strengths and limitations. The methodology allowed for exploration of heterogeneous intervention modalities and enabled us to summarise research using a variety of study designs and methodologies, with a variety of outcomes reported. As a result, this scoping review provides a broad overview of the latest published evidence investigating the use of digital interventions in prediabetes. Further, the review appears timely, given the increasing number of studies published in the last few years. However, there are also limitations to scoping reviews. While this review provides a descriptive overview of findings, a full systematic review is a recommended next step to provide a thorough analysis and appraisal of the evidence. The heterogeneity across studies, in particular with respect to study designs, intervention modalities, and outcomes, is also a limitation as it prevented in-depth analysis and synthesis of the findings. Finally, our reliance on published data subjected the review to publication bias; it is possible that real-world trials of digital interventions may have been missed.

## Conclusion

This scoping review has provided an overview of current existing evidence about digital interventions to support individuals in the self-management of prediabetes. Existing interventions, covering a breadth of study methods and design, suggest that digital interventions are acceptable and have the potential to reduce adults’ risk of progressing from prediabetes to type 2 diabetes. However, more research is needed to understand which interventions, and which components therein, have the greatest reach into diverse populations, are most effective at promoting user engagement, and are most effective in the longer term.

## Supporting information

S1 Checklist Preferred Reporting Items for Systematic reviews and Meta-Analyses extension for Scoping Reviews (PRISMA-ScR) checklist. (PDF)

S1 Table Search strategy applied to medline. (DOCX)

S2 Table Review criteria. (DOCX)

S3 Table Characteristics of included studies. BMI indicates body mass index; HbA1c, haemoglobin A1c; RCT, randomised controlled trial; CDC, Centers for Disease Control and Prevention; SMART goal-setting, Specific, Measurable, Achievable, Relevant and Time-bound goal-setting.
(DOCX)

S1 File (PDF)
