# Supplementary material for: Digital interventions for self-management of prediabetes: A scoping review
Source: PLoS One. 2024 May 10;19(5):e0303074. doi: 10.1371/journal.pone.0303074 (PMC11086829; doi:10.1371/journal.pone.0303074)
Supplement: S1 Table — (DOCX) [file pone.0303074.s002.docx]

**S1 Table.** **Search strategy applied to Medline.**

| **1** | Exp Mobile Applications/ |
| --- | --- |
| **2** | exp Internet/ |
| **3** | exp Computers, Handheld/ |
| **4** | (app or apps).ti,ab. |
| **5** | (online or web or internet or digital*).ti. |
| **6** | (prediabetes or pre-diabetes).ti,ab. |
| **7** | “(smartphone*” OR “smart phone” OR “mobile phone” OR “cellular phone” OR “cell phone” OR “mobile app*” OR “mobile device” OR “mobile-based” OR “mobile health” OR “mhealth” OR “m-health) |
| **8** | digital AND health |
| **9** | social media/ or electronic communication/ or online social networks/ or exp computer mediated communication/ |
| **10** | electronic communication/ or blog/ or text messaging/ |
| **11** | (internet or web or social media or blog* or messag* or text messag* or electronic or ehealth or telecommunication*).ti,ab. |
| **12** | 1 or 2 or 3 or 4 or 5 or 7 or 8 or 9 or 10 or 11 |
| **13** | 6 and 12 |
| **14** | Limit 13 to adults |
