# Supplementary material for: Digital interventions for self-management of prediabetes: A scoping review
Source: PLoS One. 2024 May 10;19(5):e0303074. doi: 10.1371/journal.pone.0303074 (PMC11086829; doi:10.1371/journal.pone.0303074)
Supplement: S2 Table — (DOCX) [file pone.0303074.s003.docx]

**S2 Table. Review criteria**.

| **Population** | Adults (aged 18+ years) from any country with a diagnosis of prediabetes |
| --- | --- |
| **Exposure** | Any digital intervention designed to promote self-management of prediabetes |
| **Comparator** | Any comparator or no comparator (e.g. single-arm studies) |
| **Outcome** | Any measure of health, clinical diagnoses, physical activity, weight loss, knowledge, engagement/satisfaction |
| **Study design** | Any study design (excluding protocols, reviews, and conference abstracts) published any date to December 2022 |
