# Supplementary material for: Digital interventions for self-management of prediabetes: A scoping review
Source: PLoS One. 2024 May 10;19(5):e0303074. doi: 10.1371/journal.pone.0303074 (PMC11086829; doi:10.1371/journal.pone.0303074)
Supplement: S3 Table — BMI indicates body mass index; HbA1c, haemoglobin A1c; RCT, randomised controlled trial; CDC, Centers for Disease Control and Prevention; SMART goal-setting, Specific, Measurable, Achievable, Relevant and Time-bound goal-setting. (DOCX) [file pone.0303074.s004.docx]

**S3 Table.** Characteristics of included studies

| **Authors, year** | **Aims** | **Country** | **Design** | **Population** | **Intervention** | **Control** | **Key findings** |
| --- | --- | --- | --- | --- | --- | --- | --- |
| Al-Hamdan et al., 2021 [24] | To investigate the effectiveness of educational programs among Saudi women with prediabetes | Saudi Arabia | 3-arm cluster randomised, multi-intervention and multicentre study with follow-up at 3 and 6 months post-baseline | N=120 Saudi women aged 18–60 years with prediabetes (n= 40 intervention 1; n=43 intervention 2; n=37 control) | 6 months of 1 of the following interventions:   - Intervention 1: group intensive lifestyle education group program consisting of six structured educational sessions - Intervention 2: WhatsApp education program providing the same information via social media and an app; access to testing and monitoring resources | Standard care: non-personalised advice from the primary healthcare provider including distribution of evidence-based pamphlets and booklets | At 6 months, glycated haemoglobin significantly improved in all groups, including control, post-intervention but with no difference in between-group comparisons. Significant changes in weight, lipids, and diet were only observed in the intensive educational group |
| Alwashmi et al., 2019 [25] | To investigate the effectiveness of a novel digital therapeutic diabetes prevention program | Not specified | Secondary analysis of data collection at baseline and 4 months | N=273 adults aged 18+ with prediabetes and enrolled in the Transform Diabetes Prevention Program | 12-month digital version of the CDC Diabetes Prevention Program curriculum on healthy eating and physical activity; received Fitbit, scale, health coaching and group support | None | At 4 months, participants had significant reductions in weight, BMI, and work absenteeism; significant increases in physical activity |
| Batten et al., 2022 [26] | To investigate the effects of a digital diabetes prevention intervention on weight and physical activity levels | Not specified | Secondary analysis of data collected at baseline and 12 months | N=1095 adults aged 18+ enrolled in the Virgin Pulse Transform for Prediabetes program and completed 9+ months | 12-month app-based version of CDC Diabetes Prevention Program: 4-month high frequency core intervention and 8-month maintenance for healthy behaviours; health coach messages, calls and emails; surveys and quizzes; private group support chatrooms; wearable tracking device and scale; food diary reviewed by health coach | None | At 12 months, participants demonstrated significant reductions in body weight and significant increases in physical activity |
| Block et al., 2015 [27] | To evaluate the effectiveness of a fully automated algorithm-driven behavioural intervention for diabetes prevention | United States | Randomised, wait-list controlled trial (usual care) with follow-up at 3 and 6 months post-baseline | N=339 adults aged 30-69 with clinical evidence of prediabetes (n=163 intervention; n=176 control) | Fully algorithm-driven, 1-year behavioural intervention for diabetes prevention, Alive-PD: delivered via web, internet, mobile phone, and automated phone calls; provides tailored behavioural support to improve physical activity and eating habits | Usual care: 6-month waitlist | At 6 months, intervention participants achieved significantly greater reductions than control participants in fasting glucose, HbA1c, body weight, BMI, waist circumference, triglyceride/high-density lipoprotein cholesterol, and Framingham diabetes risk score |
| Bootwong & Intarut, 2022 [28] | To test the effects of text messages for promoting physical activities in primary care patients with prediabetes | Thailand | RCT with follow-up at 8 and 12 weeks post-baseline | N=324 adults aged 35+ years with prediabetes (n=162 intervention; n=162 control) | Educational brochure about physical activity and text messages promoting physical activity, sent 5 days per week for 8 weeks | Physical activity education (brochure) alone | At 8 weeks, significant difference in physical activity measures for intervention participants; significant differences were not sustained at 12-week follow-up for physical activity energy and other secondary outcomes (weight, BMI, waist circumference, blood pressure) |
| Chen et al., 2014 [29] | To develop and test an online smart web aid for preventing type 2 diabetes | China | Quasi-cluster randomised controlled pilot study with follow-up at 6 months post-baseline | N=1022 adults aged 40-70 tested as prediabetes at participating rural village clinics | Support package for village doctors to deliver web-aided diabetes prevention. Package includes 4 cycles of lifestyle management, tailored by patient’s previous involvement in / performance on the topic. Intervention duration varied during the 6-month study period | None | Participants showed significant improvement 6 months post-baseline in vegetable intake, calorie intake, leisure-time exercises, body weight, and BMI. Significant improvements also found for self-efficacy in modifying diet, increasing physical activities, engaging relatives, knowledge about diabetes and risk factors. Most participating doctors and patients found the intervention useful and effective |
| Chen et al., 2020 [30] | To determine if mobile-app-based low-carbohydrate dietary guidance will reduce exposure to postprandial hyperglycaemia in adults with prediabetes | China | Single-blind, randomised controlled pilot study with follow-up at 3 months post-baseline | N= 100 adults aged 30-80 years with prediabetes (n=57 intervention; n=43 control) | Health management support service system and app, with 2 low-carbohydrate dietary guidance programs given over 3 months, with the first 2 weeks including flash glucose monitoring | Routine health education from a dietitian with instructions to upload dietary information and photos of each meal instead | The mobile app led to significant improvements 3 months post-baseline to postprandial hyperglycaemia, weight, BMI, percentage body fat mass, percentage of body fat, visceral fat area, triglyceride levels, dietary habits and physical activity |
| Estabrooks & Smith-Ray, 2008 [31] | To pilot test the feasibility and effectiveness of interactive voice response calls targeting physical activity and healthful eating | United States | Randomised controlled pilot study with randomisation at the class level with follow-up at 3 months post-baseline | N=77 adults with prediabetes enrolled in prediabetes prevention classes (n=39 intervention; n=38 control) | Standard care plus interactive voice response automated calls over a 12-week period with calls focused on physical activity, diet, and strategies for achieving behaviour change goals | Standard care: one-time, 90-minute diabetes prevention class | The intervention was found to be feasible, with 85% completing at least half the intervention and subsequent reports of their usefulness and functionality. Those who used the system lost approximately 3% body weight at 3-month follow-up, which approached significance when compared to controls, but the intervention did not have a detectable effect on behavioural measures of eating and physical activity |
| Everett et al., 2018 [32] | To determine the feasibility, acceptability, safety, and effectiveness of an automated, personalised mHealth platform in combination with a digital body weight scale | United States | Prospective single-arm observational study with follow-up 3 months post-baseline | N=55 adults (age requirement not specified) with prediabetes | Access to the Sweetch mobile platform which uses machine learning to present users with personalised advice to achieve recommended activity, weight reduction, and diet goals; connects with a digital body weight scale and body composition analyser | None | At 3 months, high degree of retention (86%) and acceptability of the Sweetch app. Satisfaction with the digital scale was also high. Significant improvements in weight, physical activity, BMI, and HbA1c. No significant changes for fasting glucose, blood pressure; no adverse events or safety concerns reported |
| Fischer et al., 2019 [33] | To evaluate the implementation and effectiveness of a text message support program | United States | Pragmatic study with follow-up at 12 months post-baseline | N=1518 English or Spanish-speaking adults (age criteria not specified) with prediabetes (n=285 intervention; n=1233 control) | Text message program, SMS4PreDM, which sent messages promoting lifestyle change and modest weight loss, delivered 6 days per week for 1 year | Usual care | High intervention retention (91% completion at 12 months); no significant difference between groups in frequency of achieving 3%+ weight loss goal. However, more controls gained 3%+ weight compared to intervention participants. Delivery costs were low at US$100.92 per participant |
| Fischer et al., 2016 [34] | To explore whether text message support enhances weight loss in patients enrolled in the CDC Diabetes Prevention Program | United States | Randomised clinical trial with follow-up at 6 and 12 months post-baseline | N=163 English or Spanish-speaking Adults aged 18+ with prediabetes defined as HbA1c 5.7-6.4% and BMI 25-50 kg/m^2^ (n=78 intervention; n=79 control) | Text message-augmented intervention consisting of 6 messages per week for 1 year relating to nutrition, physical activity, motivation, and requests for most recent weight. Additional invitations to motivational interviewing appointments with a health coach | Standard care including access to Diabetes Prevention Program classes and individual appointments with a nutritionist or health coach for diet support | Significantly more intervention participants achieved 3% weight loss at 12 months than control participants. HbA1c and systolic blood pressure saw modest improvements in the intervention group vs control. Stratification by language demonstrated a significant treatment effect in Spanish speakers, but not in English speakers |
| Fitzpatrick et al., 2022 [35] | To assess the effectiveness of a 12-month digital Diabetes Prevention Program for older adults with prediabetes and obesity within a large, integrated health care system, including changes in weight, HbA1c, and engagement | United States | Natural experiment study with follow-up at 12 and 24 months post-baseline | N=3904 Adults aged 65-75 years with prediabetes and obesity, defined as an HbA1c of 5.7-6.4% and BMI 30+ kg/m^2^, within past 12 months (n=472 intervention; n= 3432=control) | Digitally transformed and CDC-recognised version of the CDC’s Diabetes Prevention Program (Omada Health program), 12 months in duration. Consists of 12-month behaviour change curriculum, health coaching from lifestyle coaches, virtual small group support, electronic behavioural tracking tools for nutrition, physical activity, and weight | Patients potentially eligible but not enrolled in the intervention | Mean percentage weight loss among enrolled patients over 12 months was clinically significant, but not by 24 months. There was a significant difference in mean change in HbA1C between enrolled and non-enrolled patients over 12 months, but not by 24 months. 92% completed at least 4 weekly lessons, and 75% completed all 16 weekly lessons. 89% of those enrolled participated in the program for the full 12 months |
| Graham et al., 2022 [36] | To evaluate an alternative delivery method to the CDC Diabetes Prevention Program using conversational artificial intelligence | United States | Retrospective longitudinal study of members of the Lark Diabetes Prevention Program with data collected at 12 months post-baseline | N=414 adults aged 18+ years who had either been diagnosed with prediabetes or were at high risk (n=191 intervention; n=223 control) | CDC qualifiers who completed 4+ educational sessions over 9 months: a conversational agent that delivers 26 educational lessons on topics such as losing weight, activity, managing stress and healthy eating. Members receive a digitally connected scale that automatically uploads their weight and are encouraged to weigh weekly | CDC non-qualifiers who did not complete the required CDC lessons but provided weigh-ins at 12 months | CDC qualifiers did not differ from non-qualifiers in starting BMI but did experience significantly greater weight loss maintenance at 12 months, even when controlling for relevant factors. Average adjusted weight loss maintenance at 12 months was 5.3%, consistent with in-person and hybrid-digital modes of delivery |
| Griauzde et al., 2019 [37] | To examine the feasibility and acceptability of a mobile health intervention designed to increase autonomous motivation and healthy behaviours | United States | Parallel, 3-arm, mixed-methods randomised controlled pilot study with follow-up at 3 months post-baseline | N=69 adults with prediabetes and not enrolled in the Diabetes Prevention Program (n=24 app-only; n=22 app-plus; n=23 control) | App-only: Received the same information as the control and the mobile smartphone app. Asked to use app daily to chart health-related habits and behaviours: sleep, presence, activity, creativity, and eating. Asked to reflect on/chart alignment with values, which prompted tailored messages and health tips  App-plus: received the same information as the control, mobile smartphone app, and Fitbit devices. Asked to use Fitbit scale and activity tracker daily to self-monitor weight and physical activity | CDC information about prediabetes and evidence-based ways to decrease the progression to diabetes; list of resources/tools about monitoring diet, physical activity, and weight | At 3 months, app-plus group had significantly higher retention rates than the other groups. No significant differences observed in adherence rates in intervention groups. No significant differences found for changes in motivation scores at follow-up. Qualitative feedback provided a range of positive and negative experiences with the interventions |
| Katula et al., 2020 [38] | To determine the effectiveness of a digital Diabetes Prevention Program | United States | Single-blind RCT with follow-up at 4 and 12 months post-baseline | N=599 adults aged 19+ years, prediabetic and with elevated BMI (n=299 intervention; n=300 control) | Digital version of the CDC Diabetes Prevention Program consisting of an initial 16-week curriculum focusing on weight loss, followed by a 36-week curriculum focusing on weight maintenance. Includes digital weight scale, pedometer or use of participant’s own digital tracker, and messaging with a trained health coach | Enhanced standard care consisting of a 1-time, 2-hour diabetes prevention education class led by a health educator or graduate student | Intervention produced significantly greater reductions in HbA1c and percentage change in body weight at 12 months. A greater proportion of the intervention group achieved a clinically significant weight loss of 5%+ and more intervention participants shifted from prediabetes to normal HbA1c range |
| Kim et al., 2019 [39] | To test if an underserved, low-income population would engage in a digital Diabetes Prevention Program and successfully achieve lifestyle changes | United States | Non-randomised controlled trial using historical matched controls as comparison group with follow-up at 6 and 12 months post-baseline | N=227 English or Spanish-speaking adults aged 18-75 years with a recent blood test indicating prediabetes (n=109 matched to control group) | Year-long digital Diabetes Prevention Program that included weekly educational curriculum, human health coaching, connected tracking tools, and peer support from a virtual group. Initial intensive 16-week phase followed by 36-week maintenance phase | Historical control group matched to intervention patients on age, gender, race/ethnicity, and baseline BMI | 41% of 111 participants had more than 5% weight loss at 6 months and 37% of 104 had more than 5% weight loss at 12 months. Significant difference in the mean percentage change in BMI between the intervention and control group at 6 months. High rate of program completion and more than ½ of participants were highly engaged, completing at least 9 weekly lessons of the 16-week intensive phase |
| Lim et al., 2022 [40] | To assess a smartphone app-based lifestyle intervention | Singapore | RCT with follow-up 6 months post-baseline | N=148 adults aged 21-75 years with prediabetes (n=72 intervention; n=76 control) | Standard baseline counselling plus enrolment in the Nutritionist Buddy Diabetes program, providing education to self-monitor weight, diet, physical activity, and blood glucose levels for 6 months. Included behavioural strategy, weight logging twice weekly, and coaching from remote dietitians | Standard baseline counselling with advice on healthy food plate meal-planning principles from a research dietitian | Intervention group achieved a significantly greater weight loss and a 4.3-fold increased likelihood of achieving 5% weight loss, as compared to the control group at 6 months. The likelihood of achieving normoglycemia (HbA1c) was significantly higher in intervention group than in the control group. Changes to blood pressure and cholesterol were not statistically significant |
| Mann et al., 2016 [41] | To develop and pilot test a program to enhance primary care providers' counselling about behaviour change | United States | Pragmatic randomised controlled pilot study with follow-up at 6 months post-baseline and optional 3-month visit | N=54 adults aged 18+ years with prediabetes (n=27 intervention; n=27 control) | Randomised at the provider level; those in intervention arm invited to an electronic medical record-embedded action planning tool using SMART goal setting. Designed to help providers and patients set one concrete diet and one exercise goal during a single visit. Pedometer provided to all intervention patients | Patients of providers in control group only received printed information on prediabetes and lifestyle modifications | Those in the intervention group showed a significant increase in total daily steps compared to those in the control group at 6 months. Trend toward weight loss in the intervention compared to the control group, although no differences in glycaemic control, stage of change for diet or physical activity from baseline to 6 months |
| McLeod et al., 2020 [42] | To investigate the effectiveness of a comprehensive mobile and web-based technology program | New Zealand | Randomised, parallel-group, two-arm, single-blinded superiority trial in the primary care setting with follow-up at 12 months post-baseline | N=429 adults aged 18-75 years with pre-diabetes or diabetes (n=215 intervention; n=214 control) | Usual care plus BetaMe/Melon program over 12 months, delivered through mobile and web-based platforms. Includes individual health coaching, fortnightly resource provision, online support via closed forum, and online goal tracking (core 16-week program). 36 weeks of maintenance which included only web-based peer support and goal tracking | Usual care, which included annual checks of glycaemic control, primary care standard practices such as education and advice on lifestyle factors | HbA1c levels did not differ between study arms for the pre-diabetes group. Weight reduced slightly at 12 months for participants in both study arms, with no significant differences between arms |
| Nanditha et al., 2020 [43] | To study whether mobile phone text messages can be used to motivate and educate people to follow lifestyle modifications to prevent type 2 diabetes | India and United Kingdom | RCT with follow-up at 6, 12 and 24 months post-baseline | N= 2062 adults (35-55 years old in India; 40-74 years old in UK) with high prediabetes (n=1031 intervention; n=1031 control) | Intervention group received supportive text messages 2–3 times per week over the study period with tips, suggestions and positive reinforcement for health behaviours including goal setting, physical activity, dietary planning and strategies for lifestyle change. Messages personalised based on participant’s stage of change | All participants received personalised education and motivation about health diet and benefits of physical activity | No significant reduction in the progression to diabetes in 2 years for intervention versus control group. No significant differences in secondary outcomes |
| Sepah et al., 2015 [44] | To investigate the long-term outcomes and sustainability of an Internet-based diabetes prevention program | United States | Quasi-experimental study design with single-arm pre- and post-intervention assessment of body weight, HbA1c, and program management outcomes with follow-up at 6, 12, and 24 months post-baseline | N=220 adults aged 18+ years with prediabetes and able to engage in light physical activity | Internet-based translation of the CDC Diabetes Prevention Program. Includes small group support, personalized health coaching, a weekly curriculum, and digital tracking tools. Made up of a 16-week weight loss program, & 36 weeks to maintain weight loss. Online social network for health education lessons, track weight loss and message/call health coach. Reminded to measure weight daily via email/phone call | None | Significant decreases in HbA1c and maintained weight loss after 2 years in both groups (program starters and completers). 30% of program enrollers did not complete |
| Sepah et al., 2017 [45] | To examine long-term clinical outcomes, relationship between program engagement and clinical outcomes in a digital translation of the Diabetes Prevention Program | United States | Single-arm, non-randomised longitudinal trial with follow-up collected annually for 3 years post-baseline | N=220 adults aged 18+ with prediabetes | The program consisted of 1) 1 year of a behaviour change curriculum approved by the CDC; (2) technology-enabled tools to track nutritional intake, physical activity, and body weight; (3) personalized health coaching; and (4) small group support | None | From baseline to 3 years, those participants who completed 4+ and 9+ lessons achieved significant sustained weight loss and an absolute reduction in HbA1c with an average remission from the prediabetes range to the normal glycaemic range. Website logins and group participation were the only engagement factors predictive of weight loss at 6 months and 1 year |
| Sevilla-Gonzalez et al., 2022 [46] | To identify the barriers, feasibility, usability, and effectiveness of web platform to record lifestyle habits in subjects at risk of type 2 diabetes in a middle-income setting | Mexico | Prospective interventional study with 2 arms of intervention including lifestyle changes and lifestyle changes + metformin; follow-up at 3 months post-baseline with 4 interim visits | N=77 adults aged 18-65 years with elevated BMI and prediabetes (n=33 intervention; n=44 control) | Intervention plus metformin: access to a digital platform (phone or computer) designed to record lifestyle habits and medication use within a lifestyle change program. Intervention involved lifestyle modification in-person guidance, six visits over 3 months | Lifestyle modification in-person guidance with or without metformin but without access to the web platform | Just over half of all participants lost goal amount of 3% of body weight at 3 months. Use of platform showed significant decrease in fasting glucose, 2-hour glucose concentrations, body fat percentage and waist circumference. Clinically relevant, but not statistically significant changes included reduction in daily carbohydrate, sugar intake. Non-significant increase in daily protein intake and lean body mass in non-users of platform. |
| Signal et al., 2020 [47] | To examine the utilization patterns of a comprehensive mobile and web-based technology program for people with type 2 diabetes or pre-diabetes | New Zealand | Secondary analysis of a randomised controlled trial [42] core 16-week program and semi-structured interviews at the end of the 12-month program | N=215 adults aged 18–75 years with diabetes or prediabetes and enrolled in the BetaMe/Melon program | Process evaluation of intervention participants with data from 3 sources: mobile/web platform during 16-week active support phase; online questionnaire completed during final assessment; semi-structured telephone interviews with a subset of participants | None | Engagement trajectories were similar for participants in the prediabetes and diabetes HbA1c ranges but women, younger, and ethnic majority populations had higher rates of engagement. Usefulness was ranked from most to least: education resources, health coaches, goal tracking, online peer support. 53% agreed program easy to use. Barriers to engagement: program functionality, internet connectivity, incomplete delivery of program, motivation |
| Signore et al., 2022 [48] | To determine the feasibility and acceptability of an online self-compassion informed intervention to increase physical activity for persons with prediabetes | Canada | Explanatory mixed methods study to understand the feasibility and acceptability of a two-arm, randomised, single-blind, actively controlled, online intervention with follow-up at 6 weeks and 3 months post-baseline | N= 18 adults aged 40-74 years with prediabetes; low self-reported physical activity; below average population score on self-compassion (n=9 intervention; n=9=control) | Online 6-week program that involved a 60 minute meeting with a research assistant followed by 5 education sessions, each one week apart. These covered behaviour change topics (SMART goals, action-coping planning) followed by a self-compassion intervention | Behaviour change education only (SMART goals, action-coping planning) | Retention, instructor fidelity, safety, capacity, adherence to most of the study aspects, and acceptability by participants and facilitators all met the criteria for feasibility. Recruitment rate, process time, and adherence to home practice were below the criteria |
| Staite et al., 2020 [49] | To test the feasibility and pilot-test the main outcomes for a full-scale RCT of a web-based diabetes prevention program | United Kingdom | Parallel, two-arm, single-blind RCT with follow-up at 6 and 12 months post-baseline | N=200 adults aged 18-65 years with elevated BMI and prediabetes (n=98 intervention; n=102 control) | Smartphone app delivering a web-based diabetes prevention course with SMS texts incorporating motivational interviewing techniques and step-dependent feedback messages delivered via a wearable device over 12 months. Smartphone app consists of 22 sessions on diet, physical activity and mental resilience | The control group received the wearable technology and access to the web-based program but not the text messages | No treatment effect on weight, physical activity, HbA1c, waist circumference, waist-to-hip ratio, lipid levels, or blood pressure at 12 months. Intervention found feasible with target sample size reached in 36 weeks and retention of 86% at 6-month follow-up, but follow-up rate was higher in the control group at 12 months |
| Summers et al., 2021 [50] | To evaluate outcomes of patients prescribed a digital health intervention | United Kingdom | Single-arm pre-post intervention study with follow-up at 12 months post-baseline | N=45 adult primary care patients aged 18+ years with a confirmed diagnosis of type 2 diabetes or prediabetes; n=27 diagnosed with prediabetes | Enrolment in the Low Carb Program: access to therapeutic nutrition education modules over 12 weeks. Education is personalised to the user’s health status, age, ethnicity, and dietary preferences. Lessons are taught through videos, written content, or podcasts of varying lengths. | None | Prediabetes participants showed a statistically significant mean reduction in HbA1c, with more significant decreases among those who completed 9+ lessons at 12 months post-baseline. Other outcomes not differentiated by prediabetes versus diabetes status |
| Toro-Ramos et al., 2020 [51] | To investigate the long-term weight loss and glycaemic efficacy of a fully mobile-delivered diabetes prevention program; secondary outcomes explored program engagement | United States | Parallel RCT with follow-up at 6 and 12 months post-baseline | N=202 adults aged 18+ years with prediabetes (n=103 intervention; n=99 control) | Mobile app: Noom program using CDC Diabetes Prevention Program standards; coaches engaged individually and in groups using motivational interviewing principles. App facilitated group messaging, daily challenges, education articles, food and physical activity logging, feedback on food choices. 20 weeks of core content and up to 52 weeks of maintenance | Usual care: regular medical care including a paper-based diabetes prevention curriculum; no formal intervention | Changes in participants’ weight and BMI were significantly different at 6 and 12 months between intervention and control groups, but no difference in HbA1c. Those who completed the intervention saw significant weight loss at 6 months which was largely maintained at 12 months, compared to non-completers assigned to the intervention |
| Wong et al., 2013 [52] | To determine the efficacy of delivering text messages to provide diabetes- related information | China | A single-blinded randomised controlled pilot study with follow-up at 12 and 24 months | N=104 professional drivers with prediabetes (n=54 intervention; n=50 control) | In addition to usual care, a 2-year text message program based on theory of planned behaviour and social cognitive theory behaviour. Messages grouped under 4 themes: prediabetes and diabetes information, lifestyle modification information, social benefits of lifestyle modification, and self-efficacy enhancing statements. 3 phases with frequency of texts decreasing over time | Usual care: information booklets on prediabetes, diabetes, and health behaviour information | After adjusting for baseline characteristics in intent to treat analysis, type 2 diabetes onset was marginally lower at 12 months but showed no significant difference at 24 months compared to control. In complete case analysis, intervention participants had significantly lower odds of developing type 2 diabetes at 12 months; not maintained at 24 months. Significant mean differences for BMI in intervention group, but other markers (glucose levels, cholesterol, triglycerides) saw mixed/insignificant changes |
|  | | | | | | | |
