# Supplementary material for: Digital interventions for self-management of prediabetes: A scoping review
Source: PLoS One. 2024 May 10;19(5):e0303074. doi: 10.1371/journal.pone.0303074 (PMC11086829; doi:10.1371/journal.pone.0303074)
Supplement: S1 File — (PDF) [file pone.0303074.s005.pdf]

Request for Authorship Change(s)

Authorship change requests are subject to PLOS approval. All applicable sections of this form must be completed in full before we will review your request. Please note that per the PLOS Authorship Policy we require agreement from all authors (including those being added or removed) for any changes to a submission's author list.

PLOS does not generally consider requests to add or remove authors after editorial acceptance; any late-stage requests require approval by the journal's editorial team.

In some cases, depending on the nature, timing, and/or extent of authorship changes requested, PLOS may require verification of the updated author list and contributions by an official at the authors' institution(s). Difficulties reaching an institutional official may delay your submission's peer review or publication.

Section 1. Confirm adherence to PLOS policies

☒ Check here to confirm you have read the *PLOS* Authorship policy, and that the updated author list requested in this form complies in full with the criteria outlined in the Authorship Requirements section of our policy. Key points from the policy that pertain to authorship criteria are summarized here:

Everyone listed as an author must meet all criteria for authorship, and everyone who meets the criteria for authorship should be listed as an author.

Authorship criteria for *PLOS Medicine*:

1. Conception and design of the work, acquisition of data, or analysis and interpretation of data; and
2. Drafting the article or revising it critically for important intellectual content; and
3. Final approval of the version to be published; and
4. Agreement to be accountable for all aspects of the work

Authorship criteria for all other PLOS journals:

1. Substantial contributions to one or more of the following: conception or design of the work; acquisition, analysis, or interpretation of data; creation of new software used in the work; drafting or substantially revising the article; and
2. Approved the submitted version (and any substantially modified version that involves the author's contribution to the study); and
3. Agrees to be personally accountable for the author's own contributions AND to ensure that questions related to the accuracy or integrity of any part of the work are appropriately investigated, resolved, and the resolution documented in the literature.

Any contributions that do not meet authorship criteria should be discussed in the Acknowledgments section of the manuscript; authors are responsible for obtaining permission to name individuals in the Acknowledgments.

Payment of Article Processing Charges (APC) does not qualify for authorship.

☒ Check here to confirm that all authors (including those to be added or removed) consent to the requested changes.

☒ Check here to confirm that you have read PLOS policies on Competing Interests and Funding Disclosures. Complete the following table to describe any updates needed to your article's disclosures.

	Updates needed to the submission's disclosure statement	No change needed
**Competing Interests**		☒
**Funding**		☒

Section 2. Final manuscript information

**Manuscript number**<br>e.g., PONE-D-17-00000	PONE-D-23-15333R2
**Complete author list, in correct order**<br>Please note any equal contributors with asterisks (*) or hashes (#)	Melanie Stowell, Rosie Dobson, Katie Garner, Mirza Baig, Norma Nehren, Robyn Whittaker
**Financial Disclosure** – including any additions/deletions necessary due to the change in authorship	Funding for this project was received from a project grant from Precision Driven Health. The funders had no role in study design, data collection and analysis, decision to publish, or preparation of the manuscript.<br><br>Melanie Stowell gratefully acknowledges financial support by the Fulbright U.S. Scholar Program, which is sponsored by the U.S. Department of State and New Zealand Fulbright Commission. Its contents are solely the responsibility of the author and do not necessarily represent the official views of the Fulbright Program, the Government of the United States, or the New Zealand Fulbright Commission.
**Competing Interests** – including any additions/deletions necessary due to the change in authorship	The authors have declared that no competing interests exist.
**Acknowledgments statement**<br>Please acknowledge any removed authors if they contributed to the study in any way, as well as members of any author groups who do not meet our authorship criteria.	

Section 3. Requests to add authors

Individual author addition #1

**Full name**	Dr Norma Nehren
**Email address**	norman@tehikuhauora.nz
**Full affiliation**	Te Hiku Hauora, Kaitaia, New Zealand
**Briefly describe the reason for adding this author, and explain why they were not included on the submission previously.**	Dr Nehren contributed to the project's conceptualisation, funding acquisition, and manuscript reviewing/editing. All authors agree to this addition and Dr Nehren has no conflicts of interest to report. We apologise for our error in neglecting to include Dr Nehren as an author in our initial submission (we requested the addition in the first revision of the manuscript, as soon as we'd realised the error).

Confirm that this person contributed to **all** of the authorship criteria for the relevant PLOS journal (see above).	☒
**Tick all relevant boxes to indicate this individual's specific contributions:**	
Conceptualization	☒
Data Curation	☐
Formal Analysis	☐
Funding Acquisition	☒
Investigation	☐
Methodology	☐
Project Administration	☐
Resources	☐
Software	☐
Supervision	☐
Validation	☐
Visualization	☐
Writing – Original Draft Preparation	☐
Writing – Review & Editing	☒

Individual author addition #2 (if applicable)

**Full name**	N/A
**Email address**	
**Full affiliation**	
**Briefly describe the reason for adding this author, and explain why they were not included on the submission previously.**	

Confirm that this person contributed to **all** of the authorship criteria for the relevant PLOS journal (see above).	☐
**Specific contributions:**	
Conceptualization	☐
Data Curation	☐
Formal Analysis	☐
Funding Acquisition	☐
Investigation	☐
Methodology	☐
Project Administration	☐
Resources	☐
Software	☐
Supervision	☐
Validation	☐
Visualization	☐
Writing – Original Draft Preparation	☐
Writing – Review & Editing	☐

Author group addition (if applicable)

**Group or consortium name**	N/A
**Author who represents group**	
**Briefly describe the reason for adding this author group, and explain why they were not included on the submission previously.**	

Section 4. Requests to remove authors

Author removal #1

**Full name**	N/A
**Email address**	
**Briefly describe the reason for removing this author at this time given their stated contributions to the manuscript and/or research.**	

Which authorship criteria have NOT been met by this individual? (Refer to the criteria numbers in Section 1 of this form.)	
Are you updating the article's Acknowledgments to credit this person's contributions?	(Yes/No)
If yes, did they agree to be listed in the Acknowledgments?	(Yes/No)
If no, explain why you no longer wish to credit this individual for the work attributed to them in the Contributions listings provided previously.	

Author removal #2 (if applicable)

**Full name**	
**Email address**	
**Briefly describe the reason for removing this author at this time given their stated contributions to the manuscript and/or research.**	

Which authorship criteria have NOT been met by this individual? (Refer to the criteria numbers in Section 1 of this form.)	
Are you updating the article's Acknowledgments to credit this person's contributions?	(Yes/No)
If yes, did they agree to be listed in the Acknowledgments?	(Yes/No)
If no, explain why you no longer wish to credit this individual for the work attributed to them in the Contributions listings provided previously.	

Section 5. Requests for changes to author order

**Prior author list**	
**Requested author list**	
**Briefly describe the reason for requesting a change in author order at this time.**	
**If any authors' contributions need to be updated, provide the affected authors' names and their updated contributions listings here.** Contributions should be listed using the CRediT Taxonomy terms listed below; list all terms that apply.<br><br>If no changes are needed, enter "N/A" in the response box.	

CRediT Taxonomy for author contributions

- Conceptualization
- Data Curation
- Formal Analysis
- Funding Acquisition
- Investigation
- Methodology
- Project Administration
- Resources

- Software
- Supervision
- Validation
- Visualization
- Writing – Original Draft Preparation
- Writing – Review & Editing
